# A dataset on metal-related production activities and their socio-environmental impacts in Canada

**DOI:** 10.1038/s41597-025-06106-1

**Published:** 2025-11-19

**Authors:** Marin Pellan, Titouan Greffe, Guillaume Majeau-Bettez, Anne de Bortoli

**Affiliations:** 1 CIRAIG, Ecole Polytechnique de Montréal, P.O. Box 6079, Montréal, H3C 3A7 Québec, Canada; 2 CIRAIG, Institute of Environmental Sciences, UQAM, Montreal, UMR 5805, H2X 3Y7 Quebec, Canada; 3 LVMT, Ecole des Ponts ParisTech, Cité Descartes, 6-8 Avenue Blaise Pascal, UMR 5805, 77420 Champs-sur-Marne, France; 4 CIRAIG, Department of Strategy and Corporate Social Responsibility, ESG UQAM, C.P. 8888, succ. Centre ville, H3C 3P8 Montréal, Canada

**Keywords:** Sustainability, Environmental impact

## Abstract

Metal-related production activities are essential to the low-carbon energy transition but can generate significant social and environmental impacts that influence project success and public acceptance. The MetalliCan dataset compiles and structures data from 23 open datasets and over 150 reports from more than 40 companies in metal-related sectors, offering a high-resolution, site-specific foundation for sustainability impact analysis in Canada. It was constructed following a systematic and reproducible procedure to integrate heterogeneous data sources at the finest granularity possible and ensure traceability and interoperability. MetalliCan covers 48 commodities and 270 domestic sites including active mines, smelters and refineries, as well as advanced projects. It contains information on environmental dimensions-e.g. greenhouse gases, pollutants, water, land, material use and waste-and social dimensions-e.g. affected population, conflicts, protected lands, future water risk and climate conditions. The code and dataset are openly accessible and can be exploited for industrial ecology research, such as life-cycle assessment, material flow analysis and environmental-extended input-output, as well as criticality, social and prospective studies.

## Background & Summary

Metals play a critical role in the global transition towards low-carbon energy systems and are essential for other strategic sectors such as digital technologies and defense industries^1^. The accelerating global demand for metals^2–4^, coupled with their geographically concentrated production, has raised concerns about potential supply disruptions^5,6^. Environmental, social, and governance (ESG) stakes have emerged as pivotal considerations influencing the feasibility and acceptance of mining and refining projects^7,8^, and are increasingly recognized as significant sources of future supply risks, potentially outweighing concerns related to reserve depletion^9,10^.

As metal demand is expected to increase significantly in the coming decades, secondary production may partially alleviate supply needs after 2050 once sufficient anthropogenic stocks have accumulated^5,6^. However, the development of new extraction sites appears inevitable to meet growing demand in the short term. A detailed understanding of the associated environmental and social impacts, which vary substantially across individual production sites, is therefore crucial, even more when untapped deposits and emerging mining or processing methods are involved^11,12^. Factors such as deposit type, ore grade, mining depth, tailing management, local conditions and the mining and processing techniques employed significantly shape and size these impacts^13^. Consequently, detailed, transparent, and site-specific data are essential to accurately assess the socio-environmental implications of metal production activities, which in turn enables robust ESG risk analyses and informed management strategies.

Metal production data are available through governmental statistical agencies and geological surveys-e.g. via the United States Geological Survey (USGS)^14^ and the British Geological Survey (BGS)^15^ datasets at the global level, and by StatCan (the Canadian national statistics agency)^16^ at the national level. However, these datasets often present information in aggregated forms due to secret constraints, limiting their applicability for spatially explicit analyses. Additionally, they have been criticized for omitting essential flows such as waste rock and tailings^17^, failing to track the full fate of substances from extraction to refined metal, violating conservation of mass and potentially double-counting certain stages^18^. Confusion also arises due to lexical variation and lack of clear ‘reference points’, i.e, spatio-temporally explicit system boundaries within the material flow system, leading to ambiguities for product nature and classification (e.g., ‘crude ore’ versus ‘usable ore’)^19^. Two other major sources of data exist, each with specific limitations. First, datasets developed by the research community often provide valuable, site-specific insights, particularly regarding mine lifespans, operational timelines, and geographic distribution. Prominent examples include the MinCan dataset^20^ and Historical Data Mines^21^ in Canada, and analogous datasets in Australia^22^. Yet, these datasets generally offer limited value for quantifying contemporary and future ESG risks and localized socio-environmental impacts. Moreover, they are typically released as static, ad hoc spreadsheets, lacking a systematic structure to integrate additional dimensions or enable long-term interoperability with future potential datasets. Second, commercial databases, e.g. the widely-used S&P database comprising more than 37000 mining-related records worldwide^23^ offer global coverage of mining assets. However, its high costs, emphasis on financial and production metrics over environmental and social indicators, and the frequent embedding of critical information within unstructured text descriptions limit its usability^24^. Furthermore, recent analyses have highlighted substantial gaps within this database, estimating that up to 56% of global mining sites may not be reported, with omissions not systematically correlated with commodity type or geographic location^25^.

Canada has a long and well-documented history of metal production activities. The country adopted a Critical Mineral Strategy in 2022^26^, and has been examined in a recent International Energy Agency (IEA) analysis on land-use competition between biodiversity needs and carbon neutrality solutions^27^. Its federal system creates a dynamic governance landscape where national, provincial, and territorial governments collaboratively shape critical minerals policies^28^. While Canada is often perceived as a benchmark for responsible metal production, this reputation is largely comparative rather than evidence-based^28^. Indeed, despite its advanced ESG standards, no publicly available dataset currently consolidates contemporary data at the finest granularity possible-i.e. primarily at the facility level-on a wide range of dimensions such as metal production volumes, environmental performance, social impacts, and complementary ESG dimensions across mining and refining operations.

To address this gap, the MetalliCan dataset provides structured and spatially-explicit data on metal-related production activities, spanning the entire value-chain from mining to final metals for 48 elements across 270 sites in the ten Canadian provinces and territories producing such commodities. Designed according to the FAIR principles^29,30^, the dataset integrates environmental and social dimensions into a relational SQL structure, with the full code and data processing workflow used openly provided, enabling reproducibility, extensibility, and long-term maintenance. While currently focused on Canada, its methodological framework is designed for adaptation to other countries and metals, aligning with calls for standardized physical monitoring of mining activities^19^ and the growing needs for site-specific monitoring^25,31^. It supports transparency, traceability and cross-country comparability-key requirements for initiatives like the EU Battery Passport^32^. This dataset can be coupled with existing global resource use scenarios, such as those from the International Resource Panel^33^, as well as with advanced stochastic models^34^. It provides the essential, site-specific data needed to conduct regionalized and global assessments of metal production’s alignment with planetary boundaries (e.g., climate change, freshwater and land system change, and change in biosphere integrity) and resource governance.

## Methods

The MetalliCan dataset^35^ is constructed through a systematic and reproducible procedure designed to integrate diverse data sources at the finest granularity possible-e.g. site-specific level in priority-ensuring traceability and interoperability as recently advocated in the literature^25^. Given that many production-related, social, and environmental dimensions are inherently site-specific, the dataset follows a relational structure consistent with best practices established in comparable initiatives^36^. This approach ensures data consistency, ease of integration, and clear linkages across different datasets. Specifically, the dataset is structured into separate but interlinked tables, each dedicated to a particular type of information. Its construction follows two key steps i.e. selection and preparation of input data, followed by structuring and integration into a unified relational schema. The selection of variables within the tables is informed by previous studies and established methodologies, with particular emphasis on their suitability for life-cycle inventory (LCI) modeling, material flow accounting frameworks and ESG risk evaluation.

### Data collection and curation

Table 1 provides an overview of the input datasets used to develop MetalliCan, along with their retrieval methods, data formats and preprocessing steps. The following sections describe in detail the data sources and the underlying philosophy guiding input data selection and integration.

**Table 1:** Overview of geospatial datasets used in the MetalliCan compilation, including retrieval methods and preprocessing details.

Dataset name and URL	Retrieval method and data format	Preprocessing details
Principal Mineral Areas, Producing Mines, and Oil and Gas Fields (900A)^37^	Download, vector (CSV)	2023 dataset, removed fossil fuels and mineral facilities
Critical Minerals Advanced Projects, Mines and Processing Facilities^38^	Download, vector (CSV)	2023 dataset, removed mineral facilities
A Comprehensive Historical and Geolocalized Database of Mining Activities in Canada^20^	Download, vector (CSV)	—
Critical Minerals Mapping Initiative	Downloaded, vector (from portal)	Filtered for Canada
Global Energy Monitor (GEM) iron ore^46^ and steel^70^ tracker	Download, vector (CSV)	Filtered for Canada
By-product to host ratios^47^	Download, vector (CSV)	Filtered for Canada
National Pollutant Inventory Report (NPRI)^50^	Download, vector (CSV)	2023 dataset, filtered for NAICS 4 code 2122 and 331x
GHG from large facilities^51^	Download, vector (CSV)	2023 dataset, filtered for NAICS 4 code 2122 and 331x
ClimateTRACE mineral extraction sector^48^	Download, vector (CSV)	Filtered for Canada for 2023
Global Tailings Portal^52^	Communicated by the authors, vector (XLSX)	Filtered for Canada
Machine learning-enhanced monitoring of global copper mining areas^53^	Download, vector (SHP)	Filtered for Canada
Global Mining Footprint Mapped from High-Resolution Satellite Imagery^54^	Download, vector (SHP)	Filtered for Canada
MODIS Land Cover Data^58^	Google Earth Engine, raster	Calculated mode at point for 2021
ESA World Cover^71^	Google Earth Engine, raster	Calculated mode at point for 2021
Potential Natural Vegetation (PNV)^60^	Google Earth Engine, raster	Calculated mean at point
Aqueduct 4.0^55^	Google Earth Engine, raster	Extracted at point, for 2030, 2050 and 2080 for 3 scenarios
High-Resolution (1 km) Köppen-Geiger Maps for 1901-2099 Based on Constrained CMIP6 Projections^56^	Download, raster (TIFF)	Used the 0p0083 resolution; Extracted at point, for 3 periods and 4 SSPs-RCPs scenarios
CMIP6 statistically downscaled climate indices (CanDCS-M6)^57^	Direct download, raster (NC)	Extracted at point, 5 year interval for 4 SSPs-RCPs scenarios
World Database on Protected Areas (WDPA)^63^	Download, vector (SHP)	Filtered for Canada
Indigenous Peoples’ and Local Community Lands and Territories^64^	Download, vector (SHP)	Filtered for Canada
Areas of global importance for conserving terrestrial biodiversity, carbon and water^62,72^	Download, vector (SHP)	Used the 10km resolution dataset; Extracted at point and calculated average value in 50km buffer
Global Peatland Database^61^	Download, raster (TIF)	Extracted at point
Global Human Settlement Layer (GHSL)^65^	Google Earth Engine, raster	Summed in 10km and 50km buffer for 2025 and 2030
Environmental Justice Atlas^49^	Communicated by the authors, vector (XLSX)	Filtered for Canada

#### Selecting facilities and projects to include

The list of included facilities and projects was derived from publicly available datasets provided by Natural Resources Canada (NRCan, the department of the Goverment of Canada responsible for resources, energy, earth science and remote sensing), specifically the ‘*Principal Mineral Areas, Producing Mines, and Oil and Gas Fields*’^37^ containing 277 oil and gas facilities (out of the scope of the present work), 199 mines, 73 manufacturing sites, and ‘*Critical Minerals Advanced Projects, Mines, and Processing Facilities*.’ datasets^38^, referring to 270 projects. These datasets identify active mines, smelters, refineries, and mineral projects across Canada. To ensure a consistent focus on metal production, the first dataset was filtered to exclude facilities primarily involved in coal mining, crude oil upgrading, oil and gas extraction, and industrial mineral production (e.g., salt, potash). Similarly, potash and helium projects were removed from the project dataset. Recycling facilities, primarily related to scrap collection, were also excluded to focus exclusively on primary production. After filtering, a total of 270 sites were retained for analysis.

The core variables include facility or project name, location (coordinates, city for facilities), commodities, mining or processing techniques (i.e. open-pit vs underground mining), activity status (i.e., operating, maintenance, only available for projects) and development stage (e.g., advanced, demonstration, only available for projects). These data serve as the backbone of the MetalliCan dataset, enabling linkage to a wide range of environmental and social dimensions at the facility level.

#### Compiling and pre-processing of geospatial datasets

Geographic Information Systems (GIS) and Remote Sensing (RS) techniques have increasingly facilitated the assessment of localized environmental and social impacts and risks. In the mining context, they have been used especially to study water, land and society^39,40^. A growing number of open-access geospatial datasets from national and international agencies (*e.g*., NASA, ESA, WRI) are now easily available from public repositories and cloud-based geospatial analysis platforms such as Google Earth Engine (GEE)^41^. These resources offer globally consistent information, often at high spatial resolution, that can be directly linked to mining and processing locations. Spatial overlays with such datasets are commonly used to evaluate the interface between mining projects and their geographic context^24^. The value of spatial datasets for assessing ESG risks has been demonstrated, notably through national-scale mapping efforts which systematically compile geospatial data across environmental, social, and governance dimensions. For example, recent reviews in the Australian context highlight the potential of such integrated approaches^42^.

Building on this framework, we integrated publicly available geodatasets to capture localized risk-related and impact-related data, including both static and prospective variables for Canada. They span the following main dimensions:

- **Historical information and conflict legacy**, including records of previous ownership and documentation of potential socio-environmental conflicts.
- **Geochemical information**, including deposit and ore type.
- **Production metrics**, collected from initiatives such as the *Global Energy Monitor* which aims to provide open data on the production of metal commodities and fossil fuels, although current coverage for metals is limited to steel and iron ore.
- **Pollution and direct greenhouse gases (GHG) emissions**, derived from annually updated national pollutant release inventories and greenhouse gas reporting databases, as well as from the Climate TRACE coalition which provides globally harmonized, facility-level emissions estimates using remote sensing methods.
- **Tailings storage facilities**, including year of construction, structural design type (e.g., upstream, downstream, centerline), and hazard classification.
- **Current and prospective population exposure**, e.g. population density and total population within defined buffer distances around mining and metal-production sites.
- **Protected areas, indigenous land, peatland, and ecologically valuable lands**, i.e. spatial overlap between mining and metal-production sites and protected or sensitive land categories, assessed within multiple buffer distances.
- **Current and future water risk**, including indicators of baseline water stress, water depletion, and inter-annual variability, based on standardized global water risk datasets.
- **Current and future weather conditions and climate categories**, including daily temperature range, annual precipitation, and Köppen-Geiger climate classifications-a globally used system categorizing climates based on temperature and precipitation patterns-under different Representative Concentration Pathways-Shared Socio-economic Pathways (RCPs-SSPs) scenarios.
- **Historical and current land cover**, derived from remote sensing datasets and supplemented with assumptions on natural potential vegetation to approximate a counterfactual scenario, i.e., pre-human land cover states.
- **Land occupation footprint**, delineated through manual interpretation of high-resolution satellite imagery or machine learning techniques to estimate the spatial extent of mining and processing activities.

Table 1 summarizes the geospatial datasets used in the MetalliCan compilation, including their name, their sources URLs (e.g. Zenodo repository), retrieval methods, data format and pre-processing details. Datasets were obtained through direct downloads (e.g. CSV, SHP, TIFF, NC), GEE queries or in limited cases, author-provided files. or more rarely communicated by the authors. Preprocessing primarily involved geographic filtering to isolate Canadian records, temporal filtering for the most recent data (e.g., 2023 for NPRI and GHG datasets), and spatial calculations-such as extracting dominant land cover classes, summing population within buffers, or calculating mean conservation priorities-where applicable. Industry-specific filters, including NAICS codes 2122 (mining) and 331x (manufacturing), were applied to ensure relevance to metal production. For full reproducibility, the complete processing code, including scripts for data retrieval, filtering, and spatial analyses, is available in the GitHub repository (https://github.com/marpellan/metallican_db).

#### Extracting corporate and technical reports data

A significant portion of data relevant to metal production, such as production volume, energy and water consumption, waste generation, and social performance indicators, is reported in company disclosures and technical documentation. These sources are essential for quantifying environmental and social impacts at the facility level.

To integrate these data in MetalliCan, an extensive review of corporate reports was conducted, including annual reports, sustainability disclosures, and technical reports prepared in accordance with Canada’s National Instrument (NI) 43-101 regulatory standard. Publicly available sources were consulted directly through company websites, Canada’s capital market regulatory portal (SEDAR+), and specialized mining intelligence platforms such as Mining Data Online, Mining Technology and Mindat. The data collection focused on five categories of information:

- **Ownership**: Information on site ownership, recognizing that companies typically report only their share of production and environmental burdens.
- **Production metrics**: These metrics include ore mined, ore processed, concentrate produced, smelter feed and refined metal.
- **Reserves and resources**: Classified according to standard categories, with reserves divided into proven and probable reserves, and resources into measured, indicated, and inferred resources.
- **Energy consumption**: Energy consumption data by energy carrier, specifying if available whether electricity is grid-supplied or generated on site.
- **Environmental metrics**: Organized following reporting standards such as the Global Reporting Initiative (GRI) and the Sustainability Accounting Standards Board (SASB), covering air emissions, water use, greenhouse gas emissions, waste generation (including tailings), land use and occupation, and material use.

To assess reporting quality, a structured classification system was applied. Reporting granularity was evaluated as follows on a three-level scale, from more specific to more aggregated: i) site-specific level; ii) facility-group level, i.e. aggregation across multiple sites; iii) company level. In addition, a qualitative assessment of data quality was performed. For energy and environmental metrics, we prioritized reporting environmental flows at the facility or facility-group level. When only company level data were available, we also reported environmental intensities (e.g., emissions or energy use per unit of production) when available.

#### Structuring production data through reference points

**Fig. 1:**
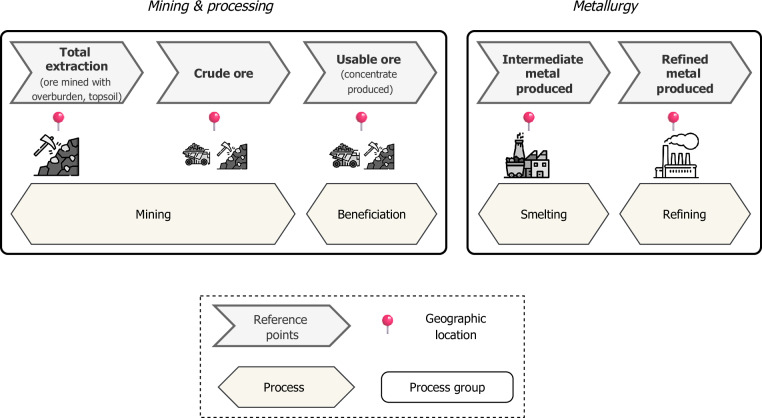
Reference points used and their relation to physical processes and physical location. Adapted from Simoni *et al*.^18^.

To ensure consistency and traceability across production metrics, a main stake when it comes to metal-related data^17,19^, we distinguish between four interconnected yet conceptually distinct levels: reference points, process groups, technical processes and physical locations. These layers are illustrated in Fig. 1, which maps how each level relates to the others within the metal product system.

Following Simoni *et al*.^18^, we define reference points as key boundaries before or after a transformation in the production process, used to anchor material flow data. They serve as checkpoints that delineate transitions between stages of the supply chain, allowing for consistent aggregation, comparison, and mass-balanced tracking of production flows. We adopt five primary reference points: *total extraction*, *crude ore*, *usable ore*, *intermediate metal produced*, and *refined metal produced*. These reference points represent the major milestones in the transformation of raw materials into final products. They are embedded within broader process groups-namely *mining*, *beneficiation*, *smelting*, and *refining*-that describe the main functional stages of metal production. Within each process group, distinct technical processes take place, such as flotation, leaching, smelting, or electro-refining, each converting material flows from one reference point to the next. While reference points are abstracted from geography and technology, they often map closely to physical locations such as the mine site, concentrator, smelter and refinery. These locations may be co-located (e.g., mine and concentrator) or spatially distinct (e.g., concentrate transported to a distant smelter or refinery).

Table 2 summarizes the definition, boundaries, production metrics, and technical attributes, i.e. performance indicators, also used as mass-balance variables, associated with each reference point. In some cases, co-existing metrics-e.g. concentrate mass and metal contained in concentrate-may co-exist at a single reference point.

**Table 2:** Summary of key reference points used for production reporting, including their definition, associated metrics, and relevant technical attributes.

Reference point	Boundaries	Production metrics	Technical attributes
Total extraction	Material extracted at the mine entrance (e.g., pit exit or underground portal), prior to transport or sorting	Total mass extracted or ore contained in mass extracted	Ore grade (% or ppm)
Crude ore	Material entering the mill or concentrator (mill feed)	Total mass processed or ore contained in mass processed	Head grade (% or ppm)
Usable ore	Output from beneficiation processes, such as flotation or leaching	Concentrate mass or contained metal in concentrate	Concentrate grade (% or ppm), Recovery rate (%), Yield (% or ppm)
Intermediate metal produced	Output from smelting processes (e.g. matte, blister metal)	Mass of intermediate metal	Smelter recovery
Refined metal produced	Final market-grade product obtained from refining, suitable for downstream use or trade.	Refined metal output	Refining recovery

This conceptual framework aimed to maximize transparency, reduce ambiguity, and ensure that material flows could be consistently traced back to clear, stage-specific reference points. However, this task was often complicated by ambiguous reporting practices and inconsistent use of units. In addition, we identified several well-documented challenges in corporate disclosures, including aggregation of data across multiple facilities-leading to the loss of spatial resolution-entailing limited availability of site-specific information, especially from major companies. Further limitations arise from ownership changes over time, which can result in reporting discontinuities and inconsistencies.

### Data integration and tables creation

#### Integrating geospatial datasets to the filtered facilities

To integrate the disparate vector datasets with our filtered list of facilities, we employed a combination of spatial and textual matching techniques using geopandas^43^ and RapidFuzz^44^. The goal was to systematically link external data to our core facility list, ensuring accuracy despite potential inconsistencies in names and coordinates across sources. We distinguished between three main types of matching depending on the nature of the data. First, for point-to-point matching, we implemented a combined approach based on spatial proximity and fuzzy name similarity. A 10 km buffer was applied following methodologies used in previous studies^45^. Name similarity was evaluated using two RapidFuzz metrics-*partial ratio* (substring match) and *token set ratio* (unordered word match)-to account for naming inconsistencies across datasets. A table of potential matches was generated for each facility, containing their geographic distance and name similarity scores. Matches were then manually inspected and validated to resolve one-to-many cases and ensure accuracy. This process was used to create definitive matching tables that link the various external datasets to our core list of facilities. This procedure was applied to the Critical Mineral Mapping Initiative (CMMI), MinCan^20^, Global Energy Monitor^46^, By-product to host^47^, Climate TRACE^48^, Conflict^49^, Pollutant^50^, GHG^51^ and Tailings^52^ datasets. Second, for point-to-polygon relationships-such as linking facilities to protected or indigenous lands-we performed spatial joins using 50 km buffers around facility locations, followed by minimum distance calculation. Third, land occupation footprints were matched to facilities using a two-step procedure. The first step involved manual name-based matching between the list of facilities drawn from the NRCan datasets and Li *et al*.^53^, which focuses on copper mining areas-although copper may not be the primary commodity at every site. his dataset provides high-resolution polygon delineations derived through machine learning techniques applied to a variety of sources, including recent Earth observation imagery. In some instances, assignments were made at the facility group level-e.g., the Sudbury operations, which span multiple facilities. The second step addressed facilities using a custom spatial assignment procedure based on datasets produced through manual interpretation of satellite imagery. Among the available sources, we prioritized the delineations from Tang & Werner^54^ due to their higher spatial resolution compared to earlier efforts such as Maus *et al*.^45^. Specifically, it provided a greater number of smaller, distinct polygons, enabling more precise spatial attribution to individual mining facilities. The assignment procedure prioritized polygons that spatially contained a facility or tailings site; when no containment was detected, the nearest polygon within a 10 km threshold was assigned. Each resulting match was categorized as one-to-one, one-to-many, or many-to-one, based on the number of polygons and entities involved. Finally, for raster-based datasets (e.g., water risk, climate data, land cover), data values were directly extracted at the exact coordinates of each facility point, as this did not require a matching process.

#### Creating structured data tables

Following the collection, curation and integration of data, tables were created and populated with relevant information. Table 3 summarizes the datasets used to construct each table.

**Table 3:** Summary of input data and their respective table in the MetalliCan schema.

Dataset	Related table(s)
Principal Mineral Areas, Producing Mines, and Oil and Gas Fields (900A)^37^	Main
Critical Minerals Advanced Projects, Mines and Processing Facilities^38^	Main
A Comprehensive Historical and Geolocalized Database of Mining Activities in Canada^20^	Main
Critical Minerals Mapping Initiative	Archetypes
Company and technical reports	Ownership, Production, Reserves, Energy, Environment flows, Environment intensity, Archetypes
Global Energy Monitor (GEM) iron ore^46^ and steel^71^ tracker	Production
By-product to host ratios^47^	By-product ratios
National Pollutant Inventory Report (NPRI)^50^	Environment flows
GHG from large facilities^51^	Environment flows
ClimateTRACE mineral extraction sector^48^	Environment flows, Production
Global Tailings Portal^52^	Tailings
Machine learning-enhanced monitoring of global copper mining areas^53^	Land occupation, Production
Global Mining Footprint Mapped from High-Resolution Satellite Imagery^54^	Land occupation
MODIS Land Cover Data^58^	Land category
ESA World Cover^59,72^	Land category
Potential Natural Vegetation (PNV)^60^	Land category
Aqueduct 4.0^55^	Water risk
High-Resolution (1 km) Köppen-Geiger Maps for 1901-2099 Based on Constrained CMIP6 Projections^56^	Climate category
CMIP6 statistically downscaled climate indices (CanDCS-M6)^57^	Weather
World Database on Protected Areas (WDPA)^63^	Protected & indigenous land
Indigenous Peoples’ and Local Community Lands and Territories^64^	Protected & indigenous land
Areas of global importance for conserving terrestrial biodiversity, carbon and water^62^	Prioritized conservation areas
Global Peatland Database^61^	Peatland
Global Human Settlement Layer (GHSL)^65^	Population
Environmental Justice Atlas^49^	Conflicts

To ensure traceability, each facility, facility-group, and reporting company was assigned a unique identifier (ID). All data entries are linked to a cited source, with each source being assigned a unique identifier and being stored in a dedicated Source table. Given the emphasis on environmental reporting, a Substance table was created to store standardized identifiers and metadata for reported environmental flows.

## Data Records

MetalliCan^35^ is organized into a relational dataset with 23 interlinked tables. It is available in SQL format, with individual tables also available in CSV format for ease of use in spreadsheet applications and simple data exploration.

The following subsections present an overview of the dataset architecture via a visual Entity-Relationship Diagram (ERD), along with detailed descriptions of the tables. This is followed by a breakdown of data coverage across key dimensions, including metals, reserves and resources, production, energy use, and socio-environmental metrics.

### Dataset structure and table descriptions

**Fig. 2:**
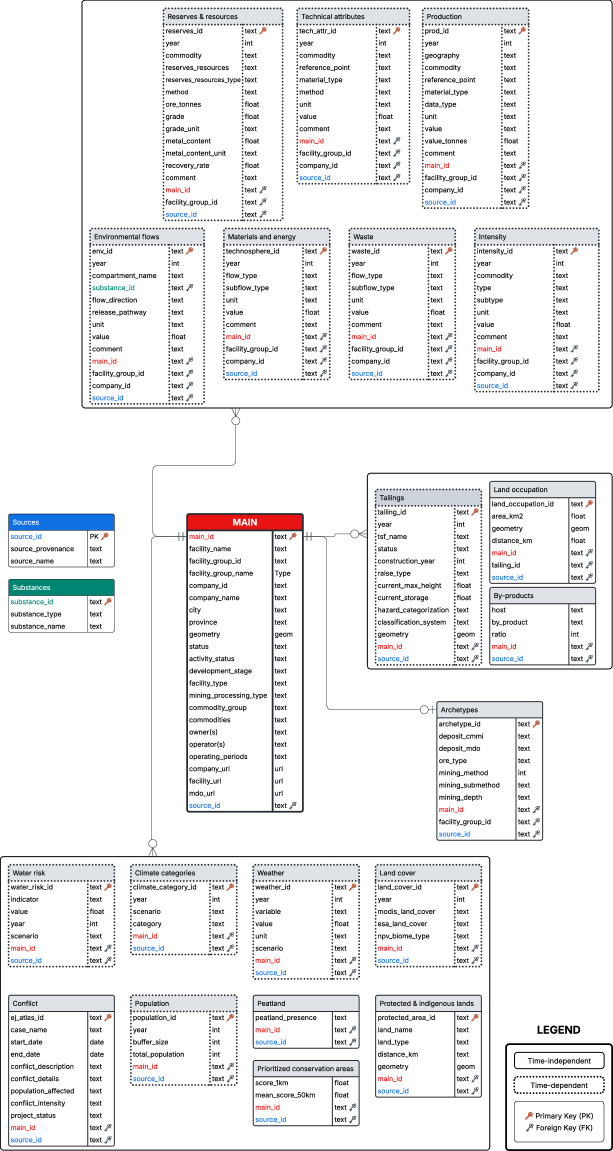
Entity-Relationship Diagram of the MetalliCan dataset. Crow’s foot notation is used for relationships involving the main table. For clarity, relationships involving the source and substance tables are not shown. The diagram was created using Lucidchart.

Figure 2 presents a simplified ERD illustrating the structure of the dataset. The diagram displays the variables in each table as well as their relationship.

Each table has its own primary key (PK) and may contain one or several foreign key (FK) for linkage with the MAIN table or one of the auxiliary tables. These FK relations are depicted using the colors of the tables in the Fig. 2.

#### MAIN table

The MAIN table serves as the backbone of the dataset, listing all known facilities and advanced projects with associated metadata. This includes mainly data from NRCan’s datasets already described, i.e. coordinates, operational status, development stage, type of facility type and reported commodities. Ownership and operator information was added from manually collected sources. When available, operating periods were supplemented using data from Dallaire-Fortin (2024)^20^.

#### Auxiliary tables

To support consistency and facilitate structured querying, two auxiliary tables, e.g. Sources and Substances provide standardized references for data provenance and environmental flows.

#### Production table

This time-dependent table records annual production data. Geographic scope is specified depending on the reporting level-i.e. Canada, or global in the case of aggregated data. Each entry includes the reference point, the material type (e.g. ore processed, contained metal in concentrate), and the data type (i.e. production volume or capacity). The original unit of measurement is retained, while a standardized transcription in metric tonnes is provided for consistency and ease of comparison.

#### Technical attributes table

This time-dependent table records technical attributes associated with mining and processing activities, including ore grade, head grade, and recovery rate. Each entry specifies the reference point and material type, ensuring compatibility and seamless integration with the Productiontable for combined analyses.

#### Reserves & resources table

This time-dependent table stores reserves and resources data, including classification (i.e., proven or probable for reserves, and measured, indicated or inferred for resources), quantities (ore, grade, metal content in ore), and corresponding units. Where available, recovery rates are also included.

#### Archetype table

This time-independent table provides information on deposit types, ore type as well as mining methods and sub methods. While not the focus of this study, this table supports future analysis by enabling the estimation of missing data based on shared attributes among similar operations.

#### Materials and energy table

This time-dependent table records material and energy inputs by type, including chemicals such as cyanide and sulfuric acid, and energy sources such as diesel, propane, explosives, and electricity. For electricity, the table distinguishes between grid-connected and on-site generation where data are available.

#### Environmental-related indicators tables

Environmental-related indicators are present in five tables.

The Environmental flows table is one of the most populated tables in the dataset. It contains reported annual quantities of GHG emissions, pollutants, water withdrawal, water consumption and water discharge. The Intensity table is similar in structure but includes aggregated or normalized environmental data-i.e., emissions per tonne of product-particularly when only company level reporting was available. The Waste table provides detailed records of mining-related waste quantities, including waste rock, overburden, and tailings. The Tailings table includes tailings facility data derived from the Global Tailings Portal^52^. It captures design type, construction year, hazard classification, and storage volume, and links each tailings site to its corresponding production facility. Finally, the Land occupation table includes polygon geometries from Li *et al*.^53^ and Tang&Werner^54^, linked to facilities, projects and tailings sites via the matching method described in the Methods section. The occupied area (in km^2^) is also reported.

#### Social and ESG-related indicators tables

Social and ESG-related indicators are distributed across 8 tables. Three tables capture current and future environmental risk. The Water risk table derived from the Aqueduct 4.0 dataset^55^ reports semi-quantitative indicators of water stress, water depletion, interannual variability, groundwater table decline, and coastal eutrophication potential. Projections are provided at five-year intervals up to 2080 under three scenarios: business-as-usual, pessimistic, and optimistic. The Climate categories table based on Beck *et al*.^56^ provides Köppen-Geiger climate classifications across four SSP-RCP scenarios (SSP1-2.6, SSP2-4.5, SSP3-7.0, and SSP5-8.5) for three time periods: 1991-2020, 2041-2070, and 2071-2099. The Weather table contains climate projection data from the CanDCS-M6 downscaled Coupled Model Intercomparison Project (CMIP) 6 dataset^57^ providing estimates of daily temperature range and annual precipitation at five-year intervals by 2100 for the same four SSP-RCP scenarios.

Four additional tables characterize land features and conservation value in the proximity of facilities and projects. The Land cover table includes land cover classification for 2021 based on MODIS^58^ and ESA WorldCover^59^ satellite products, alongside the Potential Natural Vegetation (PNV) index from Hengl *et al*.^60^, which serves as a proxy for pre-industrial land cover conditions. The Peatland table derived from the Global Peatland dataset^61^ identifies the presence and dominance of peatland areas surrounding each facility. The Prioritized conservation areas table uses data from Jung *et al*.^62^ who identified areas of global importance for biodiversity, carbon storage, and water regulation. It reports the conservation priority rank (1-100; 1 = highest priority) both at the facility location and as a mean within a 50km radius. The Protected & indigenous lands table records the name, type, and geometry of protected and Indigenous lands within a 50 km radius. It combines data from the World Database on Protected Areas^63^, the Landmark database^64^ on Indigenous Peoples and local communities lands.

Finally, the Conflict table compiles information on socio-environmental conflicts linked to mining and processing facilities, using data from the Environmental Justice Atlas^49^, while the Population table provides estimates of population size and density in buffer zones around each facility, using the Global Human Settlement Layer (GHSL)^65^ with projections available up to 2030.

### Data overview

**Fig. 3:**
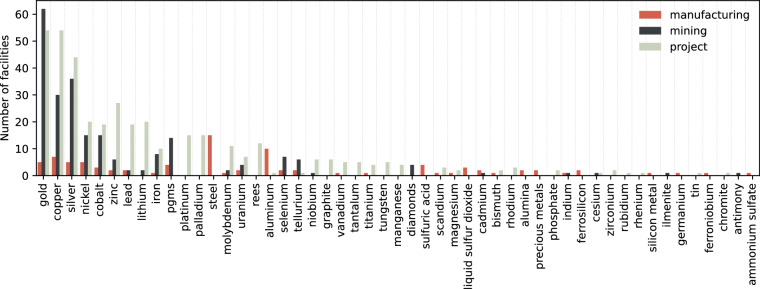
Commodities covered in MetalliCan per facility types.

Figure 3 shows the distributions of commodities covered across different facility types.

Gold, copper, and silver are the most frequently represented commodities in the dataset, associated with 121, 91, and 85 facilities, respectively. These three account for a significant portion of the total coverage. Across facility types, projects-i.e. non-producing sites-are the most common, particularly for less prevalent or emerging metals. They are followed by producing mines and manufacturing facilities, e.g. smelters and refineries.

**Fig. 4:**
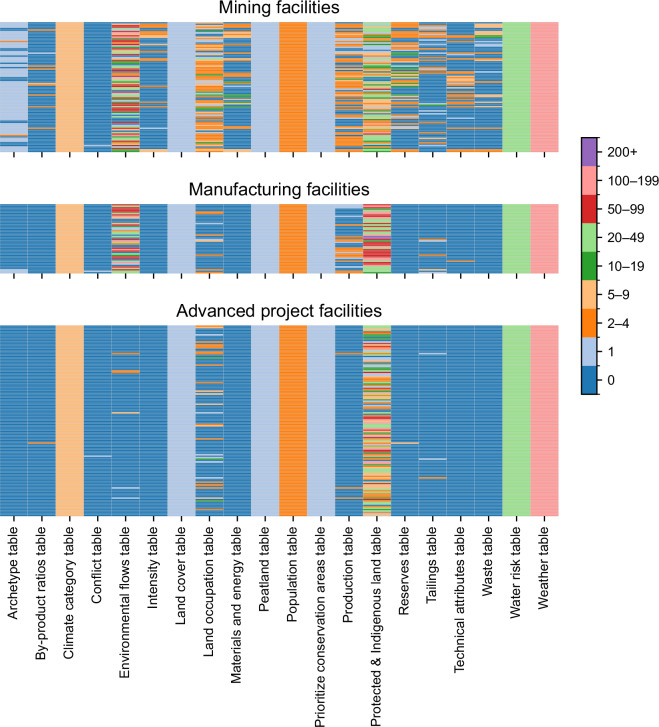
Data coverage across MetalliCan tables, shown per facility and disaggregated per facility type. The color legend indicates the number of data entries (rows) per facility in each table, grouped into value ranges to enhance visual readability.

Figure 4 displays the data coverage across tables for each facility, disaggregated per facility type.

Several patterns emerge from the heatmap. First, data coverage varies substantially across tables. Some tables are sparsely populated-e.g. the Conflict table-while others are densely filled such as the Environment, Land occupation and Protected & Indigenous land. The Climate category, Land cover, Peatland, Population, Prioritized conservation area, Water risk and Weather tables exhibit uniform coverage across all facilities. When prospective data are included-particularly in the case of temporal projections across multiple scenarios-the number of entries per facility increases. For instance, the Weather table contains data at five-year intervals up to 2100 across four SSP-RCP scenarios, as previously described. Second, data availability largely differs across facility types. Mining facilities tend to have the most comprehensive coverage, followed by manufacturing facilities-e.g., smelters and refineries. In contrast, projects typically lack data in the Production, Energy, Environment tables, reflecting their non-operational status. Third, there is considerable heterogeneity in data availability among facilities of the same type. In particular, the Environment, Land occupation, Production, Protected & Indigenous lands and Reserves tables show highly uneven coverage.

## Technical Validation

The technical validation focused primarily on the manually collected data, with a particular attention given to production volumes. For data sourced from national databases or established external datasets, we relied on the quality control procedures implemented by the original providers.

### Creating a validation dataset at national level

National-level metal production figures in Canada are reported by StatCan, notably through the *Production and Shipments of Metallic Minerals, Monthly* dataset (referred to as Table 16100019)^66^ and the *Production, shipments and value of shipments of metallic and non-metallic minerals, Yearly* dataset (referred to as Table 16100022)^16^. For commodities available in both datasets, we prioritized annual data in accordance with StatCan’s guidance. However, StatCan does not report production statistics for some commodities due to confidentiality restrictions, despite their known production within Canada. This includes, for example, cadmium, lead, niobium, and titanium. To fill these gaps, we supplemented the StatCan data with the USGS Mineral Commodities (MCS)^14^, a widely used dataset providing both global and national coverage of metals and minerals. The resulting validation sample includes 27 metals: aluminum, bauxite (alumina), cobalt, copper, diamonds, gemstones, gold, graphite, indium, iridium, iron, iron and steel, lithium, molybdenum, nickel, niobium, palladium, platinum, platinum group (as an aggregated category), rhodium, ruthenium, silicon, silver, tellurium, titanium, uranium and zinc. To enable comparison between sources and with MetalliCan, we assigned each StatCan and USGS production figure to a specific reference production point. Although StatCan and USGS do not directly report these reference points, they do describe product types (e.g., “refined”, “concentrates”, “dore”), which we systematically mapped to our defined reference points using a controlled vocabulary. Lastly, all production figures were converted into metric tonnes to ensure consistent units across datasets.

### Conducting data verification procedures within MetalliCan

We compared site-level production values with those reported by Jasansky *et al*.^36^ for sites where a match could be identified. While their data does not extend to 2023, the comparison served as a useful benchmark to detect possible human errors during data entry. Specifically, we used their mineral, commodity-referring respectively to bulk materials extracted or processed, valuable contents contained in those ores or concentrates-and capacity tables to cross-check our values.

### Comparing MetalliCan production volumes with validation dataset

We compared production volumes reported in MetalliCan with values from the national-level validation dataset for a selected number of commodities and their associated reference production points in Table 4.

**Table 4:** Comparison between production volumes reported in MetalliCan and national validation datasets.

Commodity	Reference point	Value in MetalliCan	Value in validation dataset
Aluminum	Intermediate metal produced	3,531,969	3,301,844 (StatCan); 3,200,000 (USGS)
Cobalt	Refinery production	3,368	4,923 (StatCan)
Gold	Usable ore	179	191 (StatCan); 198 (USGS)
Iron	Usable ore	80,514,117.55	82,873,581 (StatCan)
Lithium	Usable ore	33,120	31,600 (StatCan); 3240 (USGS)
Silver	Usable ore	182	241 (StatCan); 306 (USGS)

Values are given in metric tons.

The degree of alignment varies across commodities. For some metals, such as aluminum, gold, and iron, the figures from MetalliCan closely match those from the validation dataset. For others, including cobalt and silver, larger discrepancies were observed.

These differences reflect recurring challenges encountered during data collection and comparison. A central difficulty was accurately assigning the relevant reference point, as reported figures did not always clearly specify whether they referred to ore, concentrate, or metal content. Related uncertainty arose in determining the associated production metric-for instance, whether the value represented ore, concentrate, or metal in concentrate-especially when product types were not explicitly indicated. In some cases, figures may have been aggregated across production stages, making direct comparison with MetalliCan’s reference points more complex. An additional challenge involved harmonizing reported units, as production volumes were expressed in various formats such as ounces or pounds, requiring careful conversion to metric tonnes for consistent comparison. Finally, substantial variability was observed between StatCan and USGS figures for certain commodities-noticeably for silver, and even more so for lithium-even when both sources appeared to report the same reference point. These differences reflect underlying variations in data coverage, reporting methodologies, and estimation approaches across sources.

## Data Availability

The MetalliCan dataset is available at 10.5281/zenodo.17289399 and is distributed under the Creative Commons Attribution 4.0 International (CC BY 4.0) license.

## Code availability

The code used to generate and process the dataset is accessible under the BSD 3-Clause license via the GitHub repository: https://github.com/marpellan/metallican_db.

All processing scripts were developed using Python^67^, leveraging a range of scientific and geospatial libraries, including geopandas^43^, shapely^68^, scipy^69^ and rapidfuzz^44^.
